# Interaction of Bortezomib with Cell Membranes Regulates Its Toxicity and Resistance to Therapy

**DOI:** 10.3390/membranes12090823

**Published:** 2022-08-23

**Authors:** Maria João Ramalho, Stéphanie Andrade, Joana Angélica Loureiro, Maria Carmo Pereira

**Affiliations:** 1 LEPABE—Laboratory for Process Engineering, Environment, Biotechnology and Energy, Faculty of Engineering, University of Porto, Rua Dr. Roberto Frias, 4200-465 Porto, Portugal; 2 ALiCE—Associate Laboratory in Chemical Engineering, Faculty of Engineering, University of Porto, Rua Dr. Roberto Frias, 4200-465 Porto, Portugal

**Keywords:** drug–membrane interaction, anticancer drug, drug-resistance, biomembrane models, cell membranes, lipid vesicles, liposomes, membrane physical state, drug lipophilicity

## Abstract

Bortezomib (BTZ) is a potent proteasome inhibitor currently being used to treat multiple myeloma. However, its high toxicity and resistance to therapy severely limit the treatment outcomes. Drug–membrane interactions have a crucial role in drugs’ behavior in vivo, affecting their bioavailability and pharmacological activity. Additionally, drugs’ toxicity often occurs due to their effects on the cell membranes. Therefore, studying BTZ’s interactions with cell membranes may explain the limitations of its therapy. Due to the cell membranes’ complexity, lipid vesicles were proposed here as biomembrane models, focusing on the membrane’s main constituents. Two models with distinct composition and complexity were used, one composed of 1,2-dimyristoyl-sn-glycero-3-phosphocholine (DMPC) and the other containing DMPC, cholesterol (Chol), and sphingomyelin (SM). BTZ’s interactions with the models were evaluated regarding the drugs’ lipophilicity, preferential location, and effects on the membrane’s physical state. The studies were conducted at different pH values (7.4 and 6.5) to mimic the normal blood circulation and the intestinal environment, respectively. BTZ revealed a high affinity for the membranes, which proved to be dependent on the drug-ionization state and the membrane complexity. Furthermore, BTZ’s interactions with the cell membranes was proven to induce changes in the membrane fluidity. This may be associated with its resistance to therapy, since the activity of efflux transmembrane proteins is dependent on the membrane’s fluidity.

## 1. Introduction

Bortezomib (BTZ) is the gold-standard drug for treating multiple myeloma, a hematological malignancy that develops from the proliferation of neoplastic plasma cells in the bone marrow [1]. BTZ acts as a selective and reversible proteasome inhibitor, inhibiting the ubiquitin–proteasome pathway by blocking the activity of the nuclear transcription factor kappa B (NF-κB) [2]. The ubiquitin–proteasome pathway is an ATP-dependent process representing the main intracellular protein degradation pathway in eukaryotes [3], regulating several cellular processes, such as cell cycle and division, transcription, and apoptosis. Aberrant activity of the ubiquitin–proteasome pathway leads to uncontrolled cell proliferation, playing a critical role in tumor cell pathogenesis, and it has been identified as a key target for cancer therapy [4].

BTZ was the first FDA-approved proteasome inhibitor that inhibits the proteasome complex reversibly. Due to its high antitumor activity, it has been evaluated for treating other malignancies, such as glioblastoma, showing promising results in clinical trials [5,6]. However, its high toxicity and resistance development are major limiting factors for its therapeutic success [7].

Since the molecular target of BTZ—NF-κB—is an intracellular protein complex, the drug has to be able to cross cell membranes to exert its therapeutic effect, which may induce toxicity in the cell membrane [8]. The molecular interactions of BTZ with cell membranes may then regulate its bioavailability, pharmacological activity, and toxicity. Thus, this work is intended to assess if BTZ molecular interactions with cell membranes can justify its therapeutic limitations such as low oral bioavailability, toxicity, and resistance.

BTZ is currently administered by intravenous injection or subcutaneous administration [9], due to its low oral bioavailability. This proteosome inhibitor is a third biopharmaceutical class drug, with high solubility but low intestinal permeability, which limits its oral bioavailability. BTZ’s molecular interactions with cell membranes may justify its low permeability in the intestinal mucosa.

Resistance to therapy is the other major limitation of BTZ, so most patients receiving this treatment do not respond to the drug, and the ones responding to therapy eventually relapse [10]. The transmembrane protein, P-glycoprotein (Pgp), is responsible for drug efflux and has been pointed to as a major regulator of BTZ resistance, being overexpressed in several patients with myeloma [11]. Drug–membrane interactions may also be involved in BTZ’s resistance to therapy, by regulating the activity of Pgp. Lipid membranes regulate the function on transmembrane proteins, and molecular interactions with drug may affect these cell processes.

Despite the importance of understating these drug–membrane interactions, no studies evaluating the interactions of BTZ with cell membranes have been reported so far. Cell membranes are highly complex structures composed of several different components, challenging the study of drug–membrane interactions. Hence, simple in vitro lipid biomembrane models have been proposed as suitable tools to study these interactions, focusing on lipids being the main constituents of cell membranes. Since membrane lipids regulate several membrane functions, lipid-based models are often used to predict drug’s behavior in vivo. Different lipid models have been explored as in vitro biomembrane models, such as lipid monolayers, supported lipid bilayers, micelles, and liposomes [12].

Due to their 3D structure mimicking both the inner and outer leaflets of biological membranes, liposomes are the most popular lipid model [13]. These can be categorized by their dimensions and the number of concentric bilayers, and large unilamellar vesicles (LUVs) were chosen for this work, since their curvature better resembles the cell membranes [12]. The composition of the lipid model is essential to consider the composition of biological membranes. Thus, in this work, the zwitterionic 1,2-dimyristoyl-sn-glycero-3-phosphocholine (DMPC) phospholipid was chosen, since phosphatidylcholine (PC) is the main lipid constituent of human cell membranes [14]. To evaluate the influence of the bilayer composition and complexity on the drug–membrane interactions, two distinct models were proposed for this work. One contained only DMPC on its composition, and the other consisted of DMPC, cholesterol (Chol), and sphingomyelin (SM). Chol and SM are major components of the human cell membranes, and, together with transmembrane proteins, these assemble in ordered microdomains designated as lipid rafts that regulate many cell processes, such as drug efflux by protein pumps such as Pgp [15].

For these reasons, DMPC:Chol:SM models are considered more realistic and better tools to simulate biological membranes. Thus, for the more complex model (DMPC:Chol:SM), experiments were conducted at two different pH values to evaluate the effect of the BTZ’s ionization state on the drug–membrane interactions. Thus, pH 7.4 and 6.5 were used to mimic the normal blood circulation and the intestinal acidic environment, respectively. Studies have reported intestinal pH values ranging from 6.0 to 7.0 [16], therefore, an intermediate value was chosen.

Thus, the primary objective of this work was to investigate the interactions between BTZ and mimetic biomembrane models to estimate the impact of such interactions on the in vivo behavior of the drug. The partition of BTZ, its preferred localization in the bilayer, and its effect on the physical state of the membrane were investigated.

## 2. Materials and Methods

### 2.1. Materials

Bortezomib (BTZ, MW 384.24, purity ≥ 99%, Catalog No. S1013) was purchased from Selleck Chemicals (Munich, Germany). The 1,2-dimyristoyl-sn-glycero-3-phosphocholine (DMPC, MW 677.9, Catalog No. 850345P), cholesterol (Chol, MW 386.65, Catalog No. 700000P) and sphingomyelin (SM, MW 703.03, Catalog No. 860061C) were obtained from Avanti Polar Lipids (Alabaster, AL, USA). N, N, N-Trimethyl-4-(6-phenyl-1,3,5-hexatrien-1-yl) phenyl ammonium p-toluenesulfonate (TMA-DPH, MW 461.60, Catalog No. T0775), 1,6-diphenyl-1,3,5-1,3,5-hexatriene (DPH, MW 232.32, Catalog No. D208000), chloroform (Catalog No. 366927), methanol (Catalog No. 179337), and phosphate buffered saline (PBS, 10 mM phosphate buffer, 2.7 mM potassium chloride and 137 mM sodium chloride, Catalog No. P4417) were acquired from Sigma-Aldrich (Darmstadt, Germany). PBS was prepared in ultrapure water (Milli-Q Academic, Millipore, Molsheim, France) at 0.01 M (pH 7.4). For the preparation of PBS at pH 6.5, the pH was adjusted by the addition of 0.1 M HCl (Catalog No. 320331, Sigma-Aldrich, Darmstadt, Germany).

### 2.2. Preparation of LUVs

Thin-film hydration, followed by the extrusion method, was used to prepare LUVs of DMPC and DMPC:Chol:SM to be used as simple and complex biomembrane models, respectively. First, the lipids—100% DMPC or DMPC, Chol, and SM at a molar ratio of 75:15:10—were dissolved in chloroform, and the lipid film was prepared by complete evaporation of the organic solvent. To allow the self-assembly of the lipid vesicles, the lipid film was hydrated with PBS with different pHs (7.4 or 6.5) to a final lipid concentration of 5 mM. To promote vesicle size reduction, the lipid vesicles were sonicated in an ultrasonic bath for 10 min (45 kHz, Ultrasonic cleaner, VWR, Malasya) at 37 °C (above the lipids’ phase transition temperature (*T_m_*)). Multilamellar vesicles were converted to LUVs by an extrusion process (Mini-Extruder, Avanti Polar Lipids, AL, USA) with membrane filters with 100 nm pore size.

For fluorescence-quenching studies, fluorescence probes (TMA-DPH or DPH) at a probe:lipid molar ratio of 1:100 were added to the lipid solution in chloroform before drying the lipid film. The remaining protocol was conducted as described above.

To ensure that the prepared LUVs had suitable physicochemical features to be used as biomembrane models, all LUVs were characterized. The mean size, polydispersion index (PDI), and zeta potential were measured by dynamic light scattering (DLS) using a Zetasizer Nano ZS (Malvern Instruments, Malvern, UK). The LUVs showed mean sizes of 122 ± 4 and 126 ± 9 for DMPC and DMPC:Chol:SM, neutral zeta potentials, and low PDI values (<0.01) for both models as shown in Table S1 in Supplementary Materials file. No significant changes were observed for the LUVs at acidic pH (Table S1).

### 2.3. Determination of Partition and Diffusion Coefficients by Derivative UV-Vis Spectrophotometry

The affinity of BTZ for biological membranes was evaluated by determining its partition coefficient (Kp) and the logarithm of diffusion coefficient (log *D*). These values are indicators of drugs’ lipophilicity and were determined in this work by the derivative UV-Vis spectrophotometry method. As the drug molecules partition from the aqueous buffer to the LUVs bilayer, their absorbance changes allow for determining the Kp, while avoiding using physical methods to separate the aqueous and lipid phases. The second derivative is applied to eliminate the background signal originating from the light scattered by the LUVs [17].

LUVs composed of DMPC and DMPC:Chol:SM were applied to evaluate the effect of membrane composition on the BTZ’s affinity for the membranes. To assess the impact of the BTZ’s microspecies charge, PBS with different pHs (7.4 or 6.5) was used as the aqueous medium. Briefly, a solution of BTZ in PBS (final concentration of 0.4 mM) was added to increasing concentrations of LUVs (0 to 3 mM). The samples were incubated in a 96-well plate at 37 °C with medium agitation, to allow the partition of the BTZ molecules from the aqueous medium to the lipid phase. LUVs at the same concentrations without BTZ were used as the negative control. After 30 min of incubation, the absorption spectra from 200 to 400 nm were obtained for all samples (Synergy 2 Microplate Reader, BioTek, Cheadle, UK) at 37 °C.

Then, each sample spectrum was corrected by subtracting its corresponding control spectrum (LUVs without drugs at the same lipid concentration), and then the third derivative was fitted to remove the lipids background noise. The wavelength where the LUVs’ background signal is less significant was chosen (278 nm), and then a graph of the third derivative as a function of the LUVs concentration was drawn. The Kp value was calculated from the following non-linear regression [17]:

(1) $$D_{T}=D_{W}+\frac{\left(D_{m}-D_{W}\right)\;K_{P}\;\left[L\right]\;V_{m}}{1+\;K_{P}\;\left[L\right]\;V_{m}}$$

*D* is the third derivative for the absorbance values of: total BTZ (*D_T_*), BTZ found in the lipid medium (*D_m_*), and the aqueous buffer (*D_w_*). [*L*] is the LUVs’ molar concentration, and *V_m_* is the lipid molar volume. The *V_m_* values for DMPC and DMPC:Chol:SM are 0.6630 and 0.6195 L.mol^−1^, respectively.

Additionally, the logarithm of the distribution coefficient (log *D*), an indicator commonly used for ionizable molecules, was determined by the following equation:

(2) $$\log D=\log\frac{Kp}{Vm}$$

### 2.4. Determination of BTZ Location in the Membrane by Fluorescence Quenching

A fluorescence-quenching experiment was conducted to verify the preferential localization of BTZ within the lipid bilayer. Fluorescence quenching occurs when a molecule (quencher) near a fluorescent probe can decrease its fluorescence emission due to intermolecular interactions [18]. BTZ can act as a quencher if closely located to the probe. In this work, two fluorescent probes, TMA-DPH and DPH, were used.

Briefly, TMA-DPH- or DPH-labeled LUVs (500 µM) were incubated at increasing concentrations (0 to 800 µM) of BTZ dissolved in PBS. The samples were incubated in a 96-well plate at 37 °C with medium agitation, to allow the partition of the BTZ molecules from the aqueous medium to the lipid phase. After 30 min of incubation, the fluorescence intensity was measured for all samples, with 360 nm and 420 nm as excitation and emission wavelengths, respectively (Synergy 2 Microplate Reader, BioTek, UK). LUVs of different complexity, DMPC and DMPC:Chol:SM, were applied for this experiment.

The obtained fluorescence data were plotted as the probe fluorescence intensity as a function of the BTZ (quencher) concentration ([*Q*]*_m_*). [*Q*]*_m_* corresponds to the BTZ concentration that was effectively partitioned into the bilayer and was determined by the following equation [19]:

(3) $${\left[Q\right]}_{m}=\frac{K_{P}\;{\left[Q\right]}_{T}}{\left(K_{P\;}\alpha_{m}\right)+\left(1-\alpha_{m}\right)}$$

where *Q_T_* is the total used concentration of BTZ, and α*_m_* is the volume fraction of the lipid bilayer. α*_m_* was determined by the following equation:

(4) $$\alpha_{m}=V_{m}/V_{T}$$

where *V_m_* and *V_T_* are the volumes of the lipid and aqueous medium, respectively.

Then, the Stern–Volmer constant (*K_SV_*) was determined by a linear regression of the experimental data, by fitting the following equation:

(5) $$\frac{I_{0}}{I}=1+K_{SV}\;{\left[Q\right]}_{m}$$

### 2.5. Determination of BTZ Effect on the Membrane’s Thermal Behavior by DLS

The effect of BTZ on the membrane physical state was evaluated by dynamic light scattering (DLS) in terms of variations in the lipid cooperativity (*B*) and the main phase transition temperature (*T_m_*). DLS measurements were conducted to monitor changes in the mean count rate (average photons/second), in response to an increase in temperature.

DMPC and DMPC:Chol:SM LUVs were used for this experiment. Briefly, the LUVs (4000 µM) were incubated with BTZ dissolved in PBS (400 µM) and pHs of 7.4 or 6.5. The samples were incubated at 37 °C with medium agitation, to allow the partition of the BTZ molecules from the aqueous medium to the lipid phase. After 30 min of incubation, DLS measurements were performed using a temperature interval from 10.0 to 40.0 °C (Zetasizer Nano ZS, Malvern Instruments, Malvern, UK). Then, the experimental data were plotted as the normalized count rate in function of the temperature. *B* and *T_m_* values were obtained from a non-linear regression, using the following equation [20]:

(6) $$y=A_{i}+\frac{A_{f}-A_{i}}{1+10^{B\left(\frac{1}{T}-\frac{1}{Tm}\right)}}$$

where *T* is the temperature, *B* is the cooperativity, *T_m_* is main-phase transition temperature, and *A_i_* are *A_f_* are the LUVs’ mean count rate at the beginning and the end of the experiment, respectively.

### 2.6. Data Analysis and Statistical Analysis

Three replicas were conducted for each experiment, and the attained results are presented as the mean and standard deviation (SD). All graphs were prepared using GraphPad Prism software (version 9.4.0, GraphPad Inc., San Diego, CA, USA). Student’s t-test with a 95% confidence interval was employed for the statistical analysis. *p*-values lower than 0.05 were considered statistically significant.

## 3. Results

### 3.1. Estimation of BTZ’s Affinity for Biological Membranes

**Figure 1:**
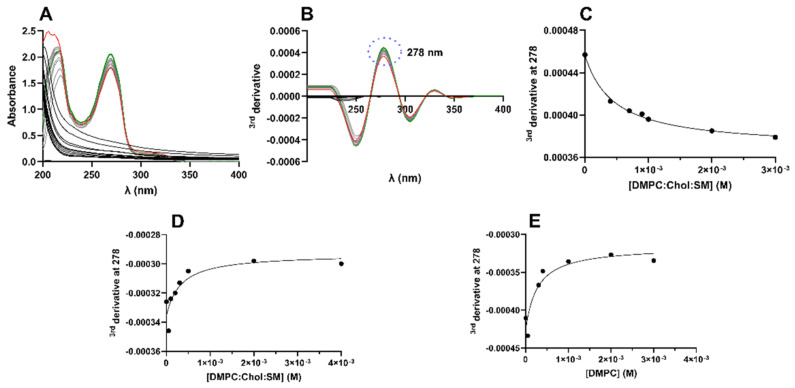
Steps for calculating the LUVs–buffer partition coefficient of BTZ. (**A**) UV–Vis spectra and (**B**) third-derivative spectra of BTZ (400 µM) at increasing concentrations of DMPC:Chol:LUVs at 37 °C and pH 6.5. The blank LUVs (control) spectra are presented in black, BTZ alone in green, and samples (BTZ incubated with increasing concentrations of LUVs) are shown in gray, with the higher concentration of LUVs presented in red. (**C**) Non-linear regression (Equation (1)) of the third derivative of experimental spectrophotometer data at λ = 278 nm for BTZ at different DMPC:Chol:SM LUVs concentrations (M). Non-linear regression (Equation (1)) of the third derivative of experimental spectrophotometer data at λ = 278 nm for BTZ at different (**D**) DMPC:Chol:SM (pH 7.4) and (**E**) DMPC (pH 7.4) concentrations (M).

LUVs were used as membrane models to determine the Kp value of BTZ, as an indicator of its lipophilicity. For the determination of BTZ’s Kp, the derivative spectrophotometry method was applied. UV–vis spectrophotometry allows for determining the Kp value, based on the assumption that the drugs’ absorption spectra changes during drug partitioning from the aqueous medium to the lipid bilayer [21]. This technique offers an advantage over the conventional separative methods, by avoiding the necessity to separate the water and lipid phases to quantify the drug on each medium after partition. The second/third derivatives of the experimental data are used to improve the resolution of the signal by eliminating the background that is generated by the light scattered from the lipids [17]. Then, the wavelength where the lipids’ background noise is negligible is chosen, and the Kp value is obtained by fitting a non-linear regression to the plotted data (Equation (1)). Figure 1A,B presents the necessary steps to determine the Kp values using this methodology. The linear regression of the obtained data for all studied models are presented in Figure 1C–E.

Then, the BTZ’s affinity for biological membranes was evaluated. For ionizable molecules such as BTZ, lipophilicity is also usually given as the logarithm of the distribution coefficient (log *D*). The higher the Kp/log *D* values, the higher the lipophilicity of the drug and, consequently, the higher the affinity for the bilayer. For each model, three independent experiments were performed. The obtained mean results are presented in Table 1.

Computational approaches are commonly used to determine drugs’ log *P* based on the octanol/water system. Thus, the predicted computational octanol/buffer log *P* value for BTZ was determined to be 1.53 (MarvinSketch software, Chemaxon, Budapest, Hungary), being very different from the experimentally obtained log *D* values. This difference was already reported for other ionizable drugs [22] and can be explained by the limitation of the octanol/water system to simulate the molecular interactions between drugs and membrane. This method is only valid for non-ionizable species, due to only considering hydrophobic interactions.

**Figure 2:**
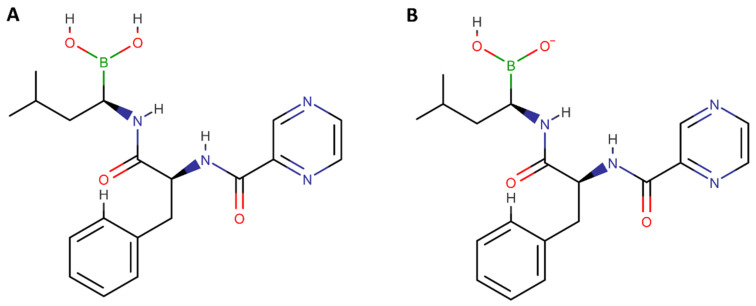
Chemical structures of the existing macrospecies of BTZ at pH (**A**) 6.5 and (**B**) 7.4 (drawn in ACD/ChemSketch).

LUVs as biomembrane models are superior to the conventional models, since these can simulate the polar forces between ionizable drugs and the phospholipids’ polar head, such as the electrostatic and ion-dipole forces. These polar interactions can also explain the different log *D* obtained for the different studied pH values. Log *D* values for DMPC:Chol:SM at pH 6.5 are significantly lower than the ones obtained for pH 7.4 (*p* < 0.05). As predicted by MarvinSketch calculator software, at pH 6.5, the majority of BTZ molecules are neutral, but at pH 7.4, a higher contribution of anionic species is verified (Figure 2). Thus, while at pH 6.5, hydrophobic interactions are prevalent, at pH 7.4, the BTZ–membrane interactions are mainly regulated by the ion–dipole and electrostatic forces between the anionic macrospecies and the lipids’ polar groups, explaining the higher obtained log *D* values for pH 7.4.

Furthermore, not only the degree of ionization of the drug influences its lipophilicity but also the composition of the membrane, as depicted by the obtained results (Table 1). LUVs with different complexities, DMPC and DMPC:Chol:SM LUVs, were applied to assess the impact of the membrane composition on BTZ’s affinity for biological membranes. In addition, the obtained results indicate that BTZ has a lower affinity for the more complex model at pH 7.4 than for the DMPC model at the same pH, as revealed by the lower log *D* values obtained for DMPC:Chol:SM (*p* < 0.05).

These complex models are more realistic models for cell membranes, and the lower affinity of BTZ can be explained by the assembly of liquid-ordered SM-Chol domains in the bilayer. This leads to an enhanced organization and increased thickness and packing of the bilayer, hindering BTZ diffusion.

### 3.2. Estimation of the Preferential Localization of BTZ in the Membrane

BTZ’s preferred location in the membrane was studied by steady-state fluorescence quenching. For that, DPH and TMA-DPH, two extensively used fluorescent probes, due to their well-known distinct locations within the lipid bilayer, were incorporated into the membrane models. TMA-DPH can be found close to the polar heads of phospholipids, and DPH is positioned deeply in the bilayer parallel to the lipids’ hydrophobic chains [23].

During its partition into the lipid bilayer, BTZ can reduce the probes’ fluorescence emission by an effect designated as fluorescence quenching. The closer the probe and the quencher, the higher the quenching effect [24]. Since BTZ’s quenching effect is dependent upon the interaction with the probe, thus, when knowing the location of the quenched fluorophore, BTZ’s preferential location can be determined.

**Figure 3:**
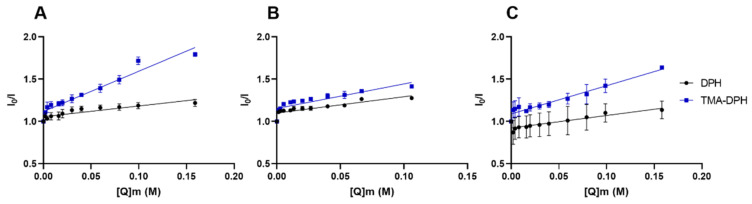
Stern–Volmer graphs showing the quenching of DPH and TMA-DPH fluorophores in the studied models: (**A**) DMPC, (**B**) DMPC:Chol:SM pH 6.5, and (**C**) DMPC:Chol:SM pH 7.4 at 37 °C, with increasing doses of BTZ.

The experimental fluorescence data in function of the BTZ concentration were plotted as Stern–Volmer graphs and presented in Figure 3. K_SV_ values were obtained from the slope of the linear regression of these graphs (Equation (5)).

K_SV_ values are indicators of the extent of the quenching phenomenon. The greater the K_SV_ values are, the greater the fluorescence intensity reduction. Thus, as BTZ acts as a quencher, the closer its proximity to the probe, the higher the quenching effect will be, yielding higher K_SV_ values. For each model, three independent experiments were performed. The attained mean results are given in Table 2.

**Figure 4:**
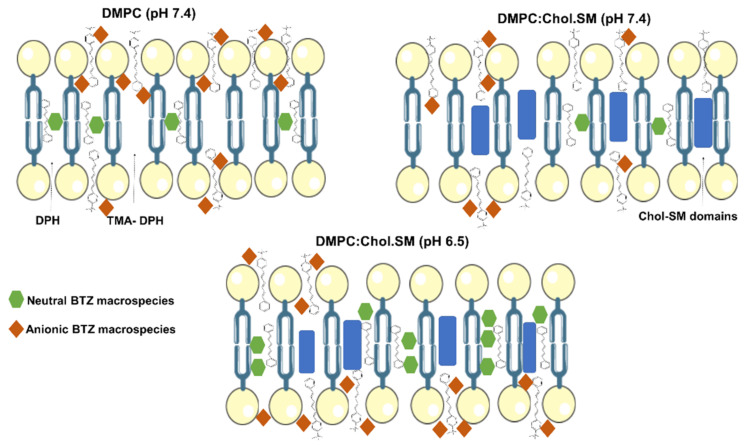
Schematic representation of anionic and neutral microspecies of BTZ with the TMA-DPH and DPH probes in the different studied LUVs models.

As depicted in Table 2, in all the used models, the BTZ’s quenching effect was higher for the TMA-DPH probe than for DPH (*p* < 0.05). The results indicate that BTZ is preferentially located close to the TMA-DPH probe, i.e., closer to the phospholipids’ polar heads. Ionizable molecules such as BTZ usually have a higher affinity for the TMA-DPH probe, as a result of the ion–dipole and electrostatic interactions between the anionic drug macrospecies and the lipids’ head groups. Additionally, it is possible to observe that the TMA-DPH K_SV_ value for DMPC:Chol:SM LUVs is significantly lower at pH 6.5 than at pH 7.4 (*p* < 0.05). The higher contribution of negatively charged microspecies at pH 7.4 can explain the obtained results, as schematized in Figure 4.

Though, the calculated K_SV_ values for the DPH also revealed that BTZ can still interact with the hydrophilic chains of the phospholipids, regardless of its ionization degree. These findings are in agreement with the determined Kp/log *D* values, showing BTZ’s high affinity for those lipid models being able to partition into the lipid bilayer and establish hydrophobic interactions with the phospholipids’ hydrophobic tails. Though, in the DMPC:Chol:SM model, this effect is more pronounced for pH 6.5, where the contribution of the charged microspecies is lower, leading to significantly higher DPH K_SV_ values for pH 6.5 than pH 7.4 (*p* < 0.05).

Finally, as observed in Table 2, the model’s complexity also affected the TMA-DPH probe’s quenching effect. Increasing the complexity of the model led to lower K_SV_ values (*p* < 0.05). This can be explained by the lower affinity of BTZ to the more complex models, as depicted by the Kp/log *D* values. Since the presence of SM-Chol domains increases the bilayers’ rigidity and packing, these decrease the drug’s partition into the biomembrane, reducing the quenching effect.

Furthermore, the obtained linear Stern–Volmer plot suggests that the quenching effect can be attributed to a dynamic phenomenon [25]. The fluorescence-quenching process can be either static or dynamic, depending on the interaction of the quencher and the probe. In a static quenching, the probe and the quencher form a stable non-fluorescent complex before excitation (ground state). On the other hand, a dynamic process, also designated as collisional quenching, happens after the collisions between the quencher and the probe [26].

### 3.3. Estimation of the BTZ’s Effect on the Membrane Fluidity and Cooperativity

DLS measurements were conducted to estimate the BTZ’s effect on the biomembranes’ physical state in terms of fluidity and cooperativity. Depending on its physical state, the membrane may exhibit distinct conformations (affecting its order, dynamics, and structure). Drug–membrane interactions often induce alterations in the cell membranes’ order, dynamics, and structure. Since these features are major regulators of cell membrane processes, the drug-induced changes are usually associated with the drugs’ toxicity.

The BTZ’s effect on the bilayer fluidity was studied in terms of variations in the lipids cooperativity (*B*) and the main phase transition temperature (*T_m_*). Depending on the temperature, the bilayer can be in a solid–gel state or in a liquid–crystalline state (more disordered state), where the phospholipids can diffuse more freely. *T_m_* is the temperature at which this phase transition occurs. Furthermore, this phase transition is a cooperative phenomenon that depends on the interaction of each phospholipid with the surrounding phospholipids. The more lipids that cooperate in the phase transition, i.e., the higher the lipids’ cooperativity, the sharper the transition will be [27].

**Figure 5:**
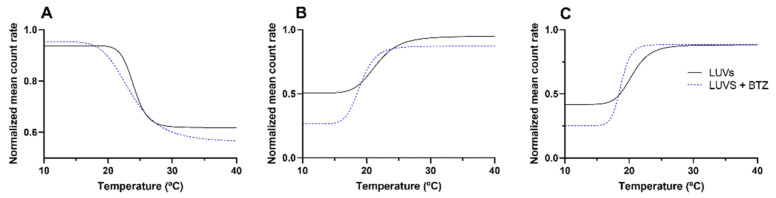
Non-linear regression (Equation (6)) of the normalized count rate with increasing temperatures with and without BTZ (400 μM), (**A**) DMPC, (**B**) DMPC:Chol:SM pH 6.5, and (**C**) DMPC:Chol:SM pH 7.4.

DLS was used to evaluate the alterations in the mean count rate as a function of temperature, as shown in Figure 5. For each model, three independent experiments were performed. The mean *T_m_* and *B* values were obtained from the non-linear regression of the obtained curves (Equation (6)). These values are presented in Table 3. The mean diameter of the LUVs was monitored during DLS measurements to confirm that these remained constant (data not shown). This ensured that the variations in the mean count rate occurred because of the macroscopic changes in the bilayer due to the phase transition.

As depicted by the obtained results, adding Chol and SM to the models affected the *T_m_* and the cooperativity of the bilayer. This effect was already reported by other authors [28].

At pH 7.4, the *T_m_* values decreased from 24.3 ± 0.2 °C for the simple model to 20.6 ± 1.3 °C for the complex DMPC:Chol:SM model (*p* < 0.05). As mentioned early, the SM-Chol domains enhance the order and rigidity of the bilayer, leading to a fluidity decrease and, consequently, lower *T_m_* values.

Regarding the lipids’ cooperativity, the *B* values decreased from 325 ± 80 for the simple model (pH 7.4) to 174 ± 16 for the complex DMPC:Chol:SM model (pH 7.4) (*p* < 0.05). The reduction in the cooperativity can be explained due to the embedding of Chol and SM molecules within the lipid bilayer. Both Chol and SM are intercalated between the phospholipids and aligned parallelly to their acyl chains. This positioning of the Chol and SM molecules reduces the number of surrounding lipids that a single lipid is able to influence [27].

Additionally, the results (Figure 5) also show that both models (simple and complex) exhibit distinct thermal behaviors. While the DMPC model exhibits a decrease in the LUVs count rate by increasing the temperature, the DMPC:Chol:SM model exhibits the opposite effect, with the mean count rate increasing with temperature for both pH 6.5 and 7.4. These results suggest that for the DMPC:Chol:SM model, the temperature increase led to the transition of the bilayer from the liquid–crystalline state (disordered phase) to the solid–gel state (more ordered state). The opposite behavior was verified for the simple model (DMPC), in which the temperature increase caused the transition from an ordered phase to a disordered state [29]. Varying the pH of the model did not affect the thermal behavior of the DMPC:Chol:SM model. Furthermore, no significant changes were verified in the *T_m_* and *B* values for the complex model at different pH values (*p* > 0.05), showing that the medium pH does not affect the bilayer fluidity.

Concerning the effect of BTZ on the membrane’s fluidity, the results showed that BTZ decreased the *B* and *T_m_* values in all the study models (*p* < 0.05), concluding that the drug can induce perturbations in the membrane. As expected, at pH 7.4, the reduction in the cooperativity was more pronounced than for the simple model (DMPC), due to the already mentioned higher affinity of BTZ for this model. In the DMPC model, the BTZ molecules can more deeply penetrate the bilayer and intercalate between the phospholipids’ tails, thus affecting their cooperativity.

Regarding the complex model (DMPC:Chol:SM), the decrease in the cooperativity and *T_m_* was more noticeable at pH 7.4 than at pH 6.5, which can be explained due to the already mentioned higher contribution of the charged macrospecies of BTZ at this value. The anionic macrospecies of BTZ can interact with the phospholipids’ head groups, decreasing the electrostatic forces between phospholipid DMPC molecules, leading to a reduction in the cooperativity [30]. Furthermore, the region near the phospholipid heads is reported to exhibit increased order and rigidity; therefore, drugs with preferential location within this region often induce more extent changes in the membrane fluidity [31].

## 4. Discussion

LUVs were used in this work as biomembrane models to estimate the impact of drug–membrane interactions on the BTZ’s bioavailability, toxicity, and resistance to therapy. It is well-established that these interactions regulate drugs’ affinity for the cell membranes and, consequently, their absorption, biodistribution, elimination, and toxicity [32]. Thus, studying BTZ–membrane interactions will allow one to better understand and estimate the drugs’ in vivo behavior.

Lipid-biomimetic-membrane models offer several advantages for studying such interactions, since, despite their simplicity, these mimic the lipid component of complex cell membranes. Lipids are the main constituents of biological membranes, providing structural stability and regulating several cell functions. No less important, the membrane lipids offer structural support for the transmembrane proteins involved in cell processes such as drug uptake and efflux [33].

Different biophysical techniques were applied to investigate different properties of BTZ and ttheir role in its interactions with the biomembrane models. The evaluated parameters were the BTZ’s lipophilicity and preferential location within the lipid bilayer, as the drug’s effect on the membrane’s physical state. The results revealed that BTZ’s interactions with cell membranes depend on its ionization degree. BTZ is an ionizable molecule, and depending on the pH value, it can exhibit different macrospecies. Thus, the interaction studies were performed at two different pH values, aiming to evaluate the effect of the BTZ’s macrospecies.

Currently, BTZ is available for intravenous injection, since its oral bioavailability is poor due to its low intestinal permeability [9]. To evaluate if these differences in BTZ’s bioavailability were regulated by its degree of ionization, the pH of these two environments was mimicked for the experiments. pH 7.4 was used to mimic normal blood circulation, and pH 6.5 was used to simulate the intestinal acidic environment.

It was verified that the degree of ionization regulated BTZ’s partition into the membrane and its preferential location within the lipid bilayer. At blood-simulated pH values, the anionic macrospecies of BTZ regulate the drug–membrane interactions by the ion–dipole and electrostatic forces with the phospholipids’ polar heads. However, at acidic intestinal pH, the contribution of the charged macrospecies is less noticeable. This explains the drug’s lower affinity for the cell membranes of the intestinal epithelium, reducing its adsorption. Thus, the obtained results suggest that the poor oral bioavailability of BTZ and its well-reported low intestinal adsorption [34] depend on its ionization degree and interaction with the intestinal cell membranes.

As usually verified in most cancers, the pH of myeloma tumor microenvironment is also more acidic than in the healthy tissues and blood circulation [35]. The characteristic acidic tumor microenvironment (pH ~ 5.6–6.8) results from the glycolysis and hypoxia in tumor cells [36]. This acidification of the tumor extracellular environment occurs due to the necessity of the cells to regulate the increased intracellular pH after tumor cells switch to anaerobic glycolysis and produce lactic acid [37]. Therefore, based on the findings of the present work, the BTZ’s uptake in the target myeloma cells might also be compromised.

Furthermore, two lipid models with distinct composition and complexity were used (DMPC and DMPC:Chol:SM) and revealed that BTZ’s interactions with the biomembranes also depend upon the model composition. Chol and SM are major components of the human cell membranes and were added to the complex model at 15% and 10%, respectively, according to studies reporting the cell membrane composition [38]. The insertion of Chol and SM into the lipid membrane increases its rigidity and packing of the bilayer, which difficult the diffusion of BTZ molecules into the bilayer. This leads to a lower affinity of BTZ to the more complex and more realistic cell membrane model.

In biological membranes, the binary mixtures of Chol and SM molecules with phospholipids assemble in ordered microdomains known as lipid rafts regulate several membrane functions and harbor transmembrane proteins [39]. Evidence has shown that these lipid rafts regulate the binding of drugs to the efflux pump Pgp involved in multidrug-resistance (MDR) [40].

MDR is one of the main causes of BTZ’s therapeutic failure and is linked to several mechanisms. Despite a few contradictory studies that report that BTZ can reduce the expression of Pgp [11], the majority of the evidence reports the overexpression of the transmembrane protein Pgp in myeloma patients and points to it as one of the major factors regulating acquired resistance to BTZ [11]. As lipid membranes have an essential role in modulating Pgp functions [41], the high resistance of BTZ may be explained by its interactions with membranes. The activity of Pgp depends on the drugs’ affinity to the membrane, since Pgp substrates only bind to the efflux pump after partitioning into the bilayer [42]. Since BTZ has a high affinity for the membrane, a great extent of BTZ–Pgp binding is expected. Additionally, as some BTZ molecules could diffuse through the membrane, avoiding the Pgp, the drug amount that effectively reaches the intracellular compartment depends on the competition between the passive diffusion and the drug efflux, as reported by other authors [41].

Furthermore, previous studies have demonstrated that Pgp activity is regulated by the lipid bilayer phase, i.e., by its fluidity and packing. In fact, recent studies have shown that its activity is higher when the bilayer is in the more disordered state (liquid–crystalline phase) [43]. The results obtained in the present study show that BTZ could induce the transition of the lipid bilayer from an ordered phase to a disordered state. The obtained finding may suggest that BTZ could enhance the efflux pump’s activity, promoting the drug resistance mechanisms. Other authors also reported that drugs’ binding affinity for Pgp also depends on the membrane composition, such as the phospholipid polar groups and the acyl chains’ length and saturation [44].

Thus, the biophysical state of the cell membranes is a key factor regulating their functions. As a result, drug-induced changes in the membrane’s physical state, such as changes in its order and fluidity, may compromise cell functions. Since BTZ must cross the myeloma cell membranes to reach its intracellular target, the NF-κB transcription factor, the drug can exert toxic effects on the membrane’s biophysical state. In fact, the thermal behavior studies showed that BTZ could exert perturbations in the membrane state, altering the *T_m_* and *B* parameters. Its toxicity towards the membranes could be related to its well-reported toxicity. BTZ’s high toxicity limits its long-term use and imposes a very narrow therapeutic window (its therapeutic dose is 1.3 mg/m^2^, while 1.5 mg/m^2^ showed toxic effects in phase I clinical trials [45]), which limits its efficacy. The most commonly reported side-effects of BTZ are peripheral neuropathy and thrombocytopenia [46].

The findings of the present work suggest that the differences in BTZ bioavailability, depending on the administration route, may result from its ionization degree and interactions with cell membranes. Further studies using more complex models such as proteoliposomes could further elaborate on the role of BTZ–membrane interactions in the Pgp-mediated resistance. Additionally, since LUVs are mimetic systems, still far from the full complexity of cellular membranes, further cell experiments could validate the obtained findings on the BTZ-induced toxicity towards the cell membranes.

As BTZ’s high toxicity and Pgp-mediated resistance severely limit its therapeutic outcomes, strategies to decrease its toxicity and circumvent MDR should be envisaged to enhance its therapeutic efficacy and safety. Most recently, the concomitant therapy using BTZ and Pgp inhibitors has been proposed to improve the effectiveness of BTZ [47]. Moreover, other drugs that modulate Pgp activity by increasing the cell membrane fluidity and permeability are being studied [48]. Another strategy to overcome these limitations is the use of nanocarriers to deliver BTZ [49].

## 5. Conclusions

Multiple myeloma accounts for 1%–2% of all cancers and is the second-most-prevalent among hematological neoplasias [50]. Despite BTZ being able to prolong patients’ survival, this malignancy is still incurable due to the limitations of current treatment [51].

Despite being often neglected, drug–membrane interactions are important factors affecting drugs’ therapeutic efficacy and toxicity. In this work, in vitro biomembrane models were used to study these interactions, aiming to further elucidate about the main limitations of BTZ associated with therapeutic failure.

Cell membranes are complex structures, therefore, the design of the biomembrane models should be carefully thought out. In this work, a simple model composed of DMPC and a more complex model close to the cell membrane’s composition produced using DMPC, Chol, and SM were applied. The findings proved that the membrane’s complexity and composition are major factors influencing the BTZ–membrane interactions. Nonetheless, this work allowed to understand better the role of the molecular interactions in the low efficacy of BTZ. BTZ’s interactions with biological membranes are mainly regulated by polar forces that increase its affinity for the cell membranes, which may facilitate its efflux by the Pgp that mediated its resistance and also induce perturbations on the cell membrane that can be related to the drugs’ high toxicity.

The resistance and toxicity of BTZ limit its therapeutic success, thus creating an urgent need for novel proteasome inhibitors with higher efficacy and safety.

## Figures and Tables

**Table 1:** Partition coefficients (K_P_) and distribution coefficients (log *D*) for BTZ in the studied biomembrane models. Data are given as mean value ± SD (*n* = 3). * *p* < 0.05 and *** *p* < 0.001 indicate a statistically significant difference when compared with DMPC LUVs (pH 7.4). ^##^ *p* < 0.01 indicate a statistically significant difference when compared with DMPC:Chol:SM LUVs (pH 6.5).

	DMPC	DMPC:Chol:SM	
	pH 7.4	pH 6.5	pH 7.4
K_P_	5832 ± 94	2265 ± 68 ***	5084 ± 128 *;^##^
log *D*	3.77 ± 0.01	3.36 ± 0.01 ***	3.71 ± 0.01 *;^##^

**Table 2:** Stern–Volmer constants (Ksv) for BTZ in the different biomembrane models. Data are given as mean value ± SD (*n* = 3). * *p* < 0.05 indicate a statistically significant difference when compared with DMPC LUVs (pH 7.4). ^#^ *p* < 0.05 and ^##^ *p* < 0.01 indicate a statistically significant difference when compared with DMPC:Chol:SM LUVs (pH 6.5). ^++^ *p* < 0.01; ^+++^ *p* < 0.001 and ^++++^ *p* < 0.0001 indicate a statistically significant difference among probes (TMA-DPH and DPH).

*K_SV_* (M^−1^)	DMPC	DMPC:Chol:SM	
	pH 7.4	pH 6.5	pH 7.4
DPH	1.26 ± 0.25	1.92 ± 0.18 *	1.47 ± 0.08 ^#^
TMA-DPH	4.75 ± 0.38 ^+++^	2.83 ± 0.06 *^,++^	3.34 ± 0.09 *^,##,++++^

**Table 3:** *T_m_* and *B* values of DMPC and DMPC:Chol:SM pH 6.5 and 7.4 in the absence or presence of BTZ. Data are given as mean value ± SD (*n* = 3). * *p* < 0.05 and *** *p* < 0.001 indicate a statistically significant difference when comparing DMPC models with DMPC:Chol:SM models. ^#^ *p* < 0.05; ^##^ *p* < 0.01; ^###^ *p* < 0.001; and ^####^ *p* < 0.0001 indicate a statistically significant difference when comparing models with BTZ incubation.

	*T_m_* (°C)			Cooperativity (*B*)		
	DMPC	DMPC:Chol:SM		DMPC	DMPC:Chol:SM	
	pH 7.4	pH 6.5	pH 7.4	pH 7.4	pH 6.5	pH 7.4
-	24.3 ± 0.2	20.5 ± 1.4 *	20.6 ± 1.3 *	325 ± 80	157 ± 21 ***	174 ± 16 ***
BTZ	23.6 ± 0.2 ^##^	19.0 ± 0.1 ^####^	18.5 ± 0.3 ^##^	104 ± 5 ^###^	112 ± 13 ^#^	109 ± 12 ^##^

## Data Availability

Not applicable.

## Supplementary Materials

The following supporting information can be downloaded at: https://www.mdpi.com/article/10.3390/membranes12090823/s1. Table S1: Physicochemical properties of the prepared LUVs. Results are gives as mean ± SD (*n* = 3).
