# Supplementary material for: Interaction of Bortezomib with Cell Membranes Regulates Its Toxicity and Resistance to Therapy
Source: Membranes (Basel). 2022 Aug 23;12(9):823. doi: 10.3390/membranes12090823 (PMC9500884; doi:10.3390/membranes12090823)
Supplement: Supplementary file 1 [file membranes-12-00823-s001.zip › membranes-1864168-supplementary.pdf]

membranes

MDPI

Supplementary material

Interaction of Bortezomib with Cell Membranes Regulates Its Toxicity and Resistance to Therapy

Maria João Ramalho [1,2,*], Stéphanie Andrade [1,2], Joana Angélica Loureiro [1,2] and Maria Carmo Pereira [1,2,*]

[1] LEPABE—Laboratory for Process Engineering, Environment, Biotechnology and Energy, Faculty of Engineering, University of Porto, Rua Dr. Roberto Frias, 4200-465 Porto, Portugal
[2] ALiCE—Associate Laboratory in Chemical Engineering, Faculty of Engineering, University of Porto, Rua Dr. Roberto Frias, 4200-465 Porto, Portugal
* Correspondence: mjramalho@fe.up.pt (M.J.R.); mcsp@fe.up.pt (M.C.P.); Tel.: +351-22-508-2262 (M.J.R.); +351-22-508-1590 (M.C.P.)

Table S1. Physicochemical properties of the prepared LUVs. Results are given as mean ± SD (n=3).

LUVs	pH	Mean size (nm)	PdI	Zeta Potential (mV)
DMCP	7.4	122 ± 4	0.09 ± 0.04	-0.3± 0.6
DMPC:Chol:SM	7.4	126 ± 9	0.08 ± 0.02	1.0 ± 0.3
DMPC:Chol:SM	6.5	123 ± 11	0.07 ± 0.01	0.5 ± 0.1
